# Individual Differences in Infants' Speech Segmentation Performance: The Role of Mother‐Infant Cardiac Synchrony

**DOI:** 10.1111/infa.70020

**Published:** 2025-04-12

**Authors:** Monica Vanoncini, Ezgi Kayhan, Birgit Elsner, Moritz Wunderwald, Sebastian Wallot, Stefanie Hoehl, Natalie Boll‐Avetisyan

**Affiliations:** ^1^ Department of Developmental Psychology University of Potsdam Potsdam Germany; ^2^ Department of Linguistics University of Potsdam Potsdam Germany; ^3^ Department of Developmental and Educational Psychology Faculty of Psychology University of Vienna Vienna Austria; ^4^ Research Focus Cognitive Sciences University of Potsdam Potsdam Germany; ^5^ Department of Psychology University of Milan ‐ Bicocca Milan Italy; ^6^ Max Planck Institute for Human Cognitive and Brain Sciences Leipzig Germany; ^7^ Institute for Sustainability Education and Psychology (ISEP) Leuphana Universität Lüneburg Universitätsallee 1 Lüneburg Germany

**Keywords:** cardiac synchrony, infant word segmentation, mother‐child interactions, recurrence quantification analysis, respiratory sinus arrhythmia

## Abstract

Caregiver‐infant coregulation is an early form of communication. This study investigated whether mother‐infant biological coregulation is associated with 9‐month‐olds’ word segmentation performance, a crucial milestone predicting language development. We hypothesized that coregulation would relate with infants' word segmentation performance. Additionally, we examined whether this relationship is influenced by the caregiving environment (i.e., parental reflective functioning) and the infant's emotional state (i.e., positive affect). Coregulation was investigated via cardiac synchrony in 28 nine‐month‐old infants (16 females) during a 5‐min free‐play with their German‐speaking mothers. Cardiac synchrony was measured through Respiratory Sinus Arrhythmia (RSA), employing Recurrence Quantification Analysis to evaluate dyadic coupling (i.e., Recurrence Rate) and dyadic predictability (i.e., Entropy). Infants' word segmentation was measured with an eye‐tracking central‐fixation procedure. A stepwise regression revealed that higher dyadic coupling, but not predictability, of the dyads' RSA was associated with infants looking longer toward the screen when listening to novel as compared to familiar test words, indicating advanced word segmentation performance (Cohen's *d* = 0.25). Moreover, cardiac synchrony correlated positively with maternal sensitivity to their infant's mental states, but not with the infant's positive affect. These results suggest that caregiver‐infant biological coregulation may play a foundational role in language acquisition.

## 1 Introduction

Coregulation (Hofer 1994) is a dynamic, nonlinear, reciprocal process in which two interacting partners, such as an infant and a caregiver, continuously influence each other's behaviors and physiological states (Bornstein and Esposito 2023). This interplay involves, for instance, infants eliciting maternal responses, while maternal actions simultaneously shape and regulate the infant's behaviors. Through this continuous exchange, the dyad creates a reciprocal feedback loop that fosters social interactions. Given its continuous shifts over time, we argue for the importance of employing a method examining dynamical nonlinear systems for studying coregulation, rather than relying on traditional linear approaches that depend on an aggregate score. While its presence may be evident at moments of infant distress (e.g., hunger, discomfort), coregulation is also present during dyadic exchanges on a moment‐to‐moment basis (Buhler‐Wassmann and Hibel 2021). The connection that children and parents feel toward one another is a consequence, at least in part, of coregulation. Infants are sensitive to the emotional content and pace of feedback from their partners already in the first days of life (Nagy 2008). Moreover, before infants acquire language, they have conversation‐like exchanges with their caregivers: they take turns in looking, touching, smiling, and vocalizing to one another (i.e., social engagement; Hobson 2002). Recent studies have found a relation between behavioral and neural aspects of coregulation (emotional facial expressions, Vanoncini et al. 2022; social gaze and infant's social brain, Vanoncini et al. 2024) and language learning. The present study aimed to examine whether this link would extend to one of the first types of coregulation, namely biological coregulation (Feldman 2007), which was measured through recursive patterns of cardiac synchrony. In addition, we examined whether the potential link between biological coregulation and infant's language learning would be related with individual factors (i.e., infant positive affect) and/or the caregiving environment (i.e., parental reflective functioning). By estimating specific contributions and interaction effects, the goal of the study was to provide new insights into the development of early biological coregulation (i.e., cardiac synchrony) in relation to language learning (i.e., word segmentation).

### 1.1 Interpersonal Synchrony and Language Development

Communication begins before infants can speak (Lipschits and Geva 2024), as seen in mother‐infant coregulation (Feldman 2012; Feldman et al. 2011). Caregivers and infants create coregulatory sequences by consistently adjusting their actions and emotional states in response to each other's current and anticipated behaviors (Fogel 1993). Caregivers adjust their behavior to match the infant's abilities and needs, which indicates parental sensitivity toward their infant. In turn, infants can learn that their own actions and emotions have meaning as they are recognized and understood by their caregiver (Atzil et al. 2018). Given the strong connection between communication and language, it is, therefore, possible that the foundation of language development partly lies in aspects of coregulation.

Coregulation is frequently indexed by synchrony or temporal interdependence of behavioral and/or biological states between individuals, which helps to sustain emotional balance (Beebe et al. 2010; Feldman 2003). There is empirical evidence for an association between interpersonal synchrony and language development. For instance, in the interaction between mothers and their 7‐ to 8‐month‐old infants, contingent vocalizations (i.e., mother to infant and infant to mother) occurred more frequently when both respiratory‐defined periods of synchrony and heart‐rate‐based attention were present (McFarland et al. 2020), which indicates that synchrony may increase infants' receptivity to language stimulation, providing a scaffolding for language development. Moreover, Vanoncini and colleagues (Vanoncini et al. 2022) found a relation between the predictability of emotional synchrony and infants' speech processing. Additionally, the predictability of maternal sensory signals, across visual, auditory, or tactile modalities, has a positive impact on infants' cognitive functions (Davis et al. 2017) and is linked with infants' social brain activity and their speech processing (Vanoncini et al. 2024).

Interpersonal synchrony has been proposed to enhance the overall parent‐infant interaction and to promote a conducive environment for language development (Harrist and Waugh 2002). Fluent and connected (i.e., back‐and‐forth) verbal and nonverbal communication between caregiver and toddler predicted later language development above and beyond other variables, such as amount or quality of parent talk (e.g., lexical diversity), and sensitive parenting (Masek, Paterson, et al. 2021). Similarly, interventions aiming to increase contingent interactions, namely prompt and meaningful exchanges, between caregivers and infants enhance language development (e.g., McGillion et al. 2017). Moreover, turn‐taking, which is positively associated with infants' brain maturity and later vocabulary size (Nguyen et al. 2023), relates to neural synchrony between mothers and their infants and children (Nguyen, Schleihauf, et al. 2021). These findings suggest that coregulated synchronized social interactions have an impact on boosting communication effectiveness and fostering a conducive environment for language growth. However, the biological mechanisms underlying the link between interpersonal synchrony and language development remain largely unexplored, presenting an exciting frontier for future research. The current study examined this by focusing on caregiver‐infant cardiac synchrony and its links with one of the milestones for infants' language development, that is word segmentation.

Word segmentation refers to the ability to perceive words in the speech signal, which is continuous and does not contain any clear‐cut cues to where words begin and end. Word segmentation may involve understanding syllable structure and patterns in spoken language to discern where one word ends and another begins (Cole and Jakimik 1980). Infants need to acquire this ability as it prepares word acquisition. Following findings that already newborns are sensitive to language‐specific rhythm information in speech (Gasparini et al. 2021 for a meta‐analysis), and that speech often contains rhythmic cues to word boundaries, as in English, where words often begin with a strong syllable, Jusczyk et al.’s (1999) seminal study set out to test whether infants would rely on rhythm information for word segmentation. They familiarized 7.5‐month‐old American English‐learning infants with two bisyllabic words with strong initial syllables and found that infants subsequently listened longer to text passages that did (vs. did not) include those familiarized words. In another experiment of the same study, the 7.5‐month‐olds recognized target words from familiarized passages as indicated by their longer looking times to target than novel words during the test phase. However, only at 11, but not at 7.5 months, infants were able to recognize target words with stress on the final syllable, a rhythmic pattern that is untypical for English. These findings of infants' use of rhythm cues to word segmentation were expanded to infants learning other languages such as German (Bartels et al. 2009; Marimon et al. 2022; Zahner et al. 2016), the native language of the infants in the present study.

Across studies and languages, infants showed either familiarity (Bartels et al. 2009) or novelty preference (Zahner et al. 2016) as a marker of word segmentation. Familiarity preference has been related to short familiarization times (Hunter et al. 1983) and younger age/less developed language (DePaolis et al. 2016); whereas, novelty preference has been related to low stimulus complexity (Kidd et al. 2012). Therefore, segmentation performance can be assessed in two ways: one evaluates the absolute difference observed novel versus familiar trials (Kirkham et al. 2002), while the other suggests that novelty preference indicates a more advanced developmental stage than familiarity preferences (Hunter and Ames 1988). Previous studies using the second approach suggest that infants with advanced babbling skills (Hoareau et al. 2019), higher later word knowledge (Singh et al. 2012), less rhythmic movement (Boll‐Avetisyan et al. 2024), greater emotional synchrony with their mothers (Vanoncini et al. 2022), and mothers with less predictable social gaze (Vanoncini et al. 2024) show a novelty preference, indicating more advanced speech segmentation skills. These findings show infants' sensitivity to rhythmic patterns in speech guides word segmentation, with individual developmental differences linked to a number of factors. Yet, the role of coregulating physiological rhythms in caregiver‐infant exchanges on word segmentation remains unexplored.

At the biological level, physiological coregulation can be measured by synchrony between caregivers' and their infants' heart rate (henceforth: cardiac synchrony) and their respiratory patterns. Both these types of physiological synchrony, their underlying mechanisms, and their implications for infant development have recently gained much attention in social development studies (DePasquale 2020). According to the biobehavioral framework of attachment, parent‐child physiological synchrony partly reflects the quality of relationships, and of coordinated behavioral and emotional responsivity (Feldman 2012). Parents and infants' interactions follow certain routines (e.g., breastfeeding routine), which are characterized by a degree of rhythmicity and regular cycles of coregulation (Montirosso and McGlone 2020). These schemas are repeated over time, allowing infants to form expectations regarding their caregiver. During early infancy, mothers and their 3‐month‐old infants exhibit coordinated heart rate patterns during face‐to‐face interactions (Feldman et al. 2011). A recent meta‐analysis showed that mother‐child cardiac synchrony is disrupted in high‐risk contexts, such as clinical difficulties, history of maltreatment, and socioeconomic disadvantage (Miller et al. 2022). Overall, these studies highlight the importance of mother‐infant physiological synchrony for understanding the physiological and social processes involved in early parent‐infant interactions.

### 1.2 Cardiac Synchrony

Cardiac synchrony can be assessed using different physiological signals (Palumbo et al. 2017). This study focused on the parasympathetic nervous system, which has been found to dynamically respond to social and emotional experiences. Specifically, we studied the coregulation in mother's and infant's Respiratory Sinus Arrhythmia (RSA), which refers to the variability in heart rate synchronized with respiration, with heart rates shortening during inhalation and prolonging during exhalation (Yasuma and Hayano 2004). RSA primarily reflects parasympathetic activity (Camm et al. 1996) by regulating arousal through the ‘vagal brake’, which slows heart rates. RSA's main advantage is that it allows to control for differences in heart‐rate frequencies across age groups, such as infants and adults (Abney et al. 2021).

According to the polyvagal theory, increased vagal influence (i.e., maintaining or increasing RSA) during interpersonal interactions reflects social engagement (Porges 2007). Cardiac synchrony might depend on the caregiver, with mothers synchronizing with their child's self‐regulation and fathers responding to shared positive affect (Lunkenheimer et al. 2021). This paper examined the overall level of RSA coregulation between mothers and their 9‐month‐old infants across a 5‐min face‐to‐face interaction. Specifically, RSA coregulation was indexed by two metrics resulting from recurrence quantification analysis (i.e., CRQA): cross‐Recurrence Rate (i.e., cross RR), namely the degree to which the two RSA trajectories tend to visit similar states; and Entropy (i.e., ENTR), which captures the predictability of the recurrence between the two RSA trajectories.

Caregiver‐infant synchrony might also be related to mothers' ability to adjust to their infant's behaviors. Cardiac synchrony indicates interpersonal connection, social engagement, and co‐regulation. By the end of the first year, coregulation patterns between mothers and infants become predominantly symmetrical (i.e., both are involved in mutual and active engagement) (Evans and Porter 2009), and almost exclusively symmetrical by the second year (Aureli and Presaghi 2010). It has been suggested that, during the first year, mother‐infant coregulation is mainly driven by the mother, reflecting her ability to adjust to her infant's behavior (Aureli et al. 2022). Among the parental mentalization constructs influencing coregulation, we focused on parental reflective functioning, defined as the parent's ability to consider the infant as a psychological agent and keep the child's mental state in mind (Slade 2005). Higher parental reflective functioning has been associated with adequate caregiving and a higher probability of secure attachment (for a review, see Camoirano 2017). This capacity allows parents to attune to their child's emotional states, facilitating effective coregulation and leading to a sense of connection. Therefore, the present study aimed to examine the role of parental reflective functioning (measured by the Parental Reflective Functioning Questionnaire; Luyten et al. 2017) as a potential moderator in the link between cardiac synchrony and infant word segmentation. Cardiac synchrony might be especially adaptive when the caregiver is empathic and has high reflective functioning, but might rather indicate high levels of stress contagion between infant and caregiver when this is not the case. An empathic caregiver should be able to down‐regulate both their own and the infant's arousal, which can contribute to infant learning and communication skills. Overall, it is well known that sensitive responsiveness is linked with child language (for a meta‐analysis, see Madigan et al. 2019), which opens up the possibility that parental reflective functioning modulates the potential association of mother‐infant cardiac synchrony and language development.

Synchrony between interactive partners can be related to their emotions. Nonverbal behavioral synchrony is positively associated with positive affect (Tschacher et al. 2014). Moreover, positive emotions are beneficial for learning as they broaden attention and enhance flexibility (Fredrickson 1998). Remarkably, cardiac synchrony increases during moments of behavioral synchrony (i.e., when two persons simultaneously show positive affect or positive vocalizations), suggesting a connection between physiological and social processes (Feldman et al. 2011). In contrast, a few studies show that mother‐infant dyads show greater cardiac synchrony when co‐regulation is needed, for instance, when the infant is distressed and presents negative affect (Nguyen, Abney, et al. 2021; Wass et al. 2019). Given the research showing how affect might modulate interpersonal synchrony, the current study examined the overall amount of infants' positive affect and its role as a moderator between cardiac synchrony and language development.

### 1.3 Current Study

We aimed to investigate whether mother‐infant coregulation at the cardiac level was associated with 9‐month‐olds’ language development. In addition, we examined the role of two potential moderators, namely parental reflective functioning and infants' affect, in this relation. We tested 9‐month‐olds, because, at that age, infants do not only increasingly coordinate social attention with mothers, evidenced, for instance, through joint attention (Carpenter et al. 1998), but they also develop their ability to segment words from speech. Given that behavioral and neural mother‐infant coregulation is associated with infants' word segmentation (Vanoncini et al. 2022, 2024), we expected that biological coregulation, here measured via cardiac synchrony, indexed by high cross RR and low ENTR, would be related to infants' ability to segment words from speech at 9 months of age. Advanced word segmentation performance would be indicated by infants looking on the screen while listening to novel, as opposed to familiar words, or vice versa. Moreover, we hypothesized that the link between cardiac synchrony and infant's word segmentation would become stronger when parental reflective functioning is higher. Finally, for infants' positive affect, we did not predict a specific direction as to whether the link between cardiac synchrony and infant's word segmentation would become stronger with a higher or lower degree of positive affect. In a stepwise regression with forward selection, we incrementally increased the models' complexity to understand the effects of the fixed effects (i.e., cardiac synchrony, parental reflective functioning, infants' positive affect) on the word segmentation performance. The hypotheses and the analysis plan were pre‐registered (see https://aspredicted.org/KHR_36Y).

## 2 Methods

### 2.1 Participants

We recruited infants and their mothers in Austria from a database of parents, originally recruited in neonatal units at local hospitals, through social media, and in mother‐child activity classes. Participants took part in a larger study for which they completed seven tasks related to different studies (Tünte et al. 2024; Vanoncini et al. 2022, 2024). Here, we used data from a face‐to‐face interaction, during which cardiac synchrony and infant's affect were measured, and a word segmentation task. Tasks' order was counterbalanced. After the testing, mothers filled in an online version of the questionnaire about parental reflective functioning, and they could do so in the lab or at home.

The sample to be collected was preregistered, but not the final sample to be included after drop‐outs. Specifically, we stated in AsPredicted #46693 that our goal was to test 90 infants, but that we would stop testing by May 2021 if we faced enrollment restrictions due to the Covid‐19 pandemic. Here, the final sample consisted of 28 9‐month‐old infants (*M =* 299.25 days, *SD* = 13.01, 16 females) and their German‐speaking mothers (*M =* 33.22 years, *SD* = 4.85), 17 of which were also included in the sample in Vanoncini et al. (2022) and 14 (for Analysis I) and 26 (for Analysis II) in Vanoncini et al. (2024). The final sample was in line with recent studies on adult‐infant synchrony (e.g., Atzil et al. 2011; Piazza et al. 2020). Data from 53 additional dyads were excluded due to not performing one or both tasks (*n* = 12), technical issues (*n* = 9), noisy cardiac data for which R‐peaks could not be detected (*n* = 18), fussiness (*n* = 6), or infants not providing a minimum of six test trials in the word segmentation task (i.e., three in each condition, *n* = 8). All included infants were predominantly exposed to German, born full term (> 37 weeks of gestation) and healthy (10‐min APGAR Score > 9; six mothers did not report the APGAR scores). Concerning the mothers' education, 20 had at least one university degree, five completed technical or high school, two obtained vocational training. Two mothers did not provide this information. In the Austrian context, it is culturally inappropriate and invasive to ask information about race/ethnicity or SES. The study was conducted in accordance with the guidelines laid down in the Declaration of Helsinki, with written informed consent obtained from a parent for each child prior to any assessments or data collection. All procedures were approved by the Ethics Committee of the University of Vienna. Participants received travel cost reimbursement and either a 15€ voucher or a children's book for their participation.

### 2.2 Materials and Procedure

#### 2.2.1 Face‐To‐Face Interaction: Cardiac Synchrony and Affect

**FIGURE 1:**
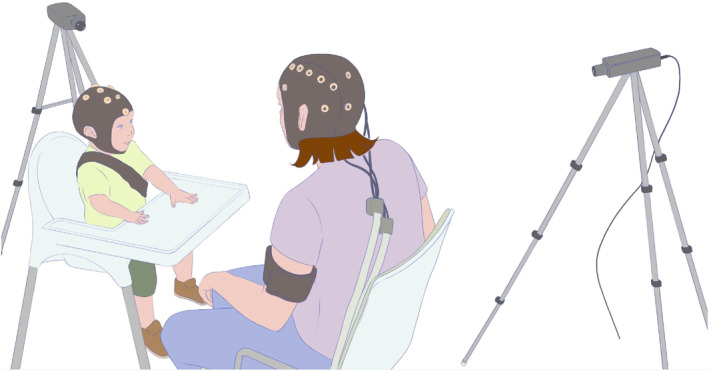
Experimental set‐up. Illustration of the experimental placement of mothers and their infants: dual electrocardiography was obtained via ECG electrodes placed underneath the T‐shirt, and the Smarting device in the bags on the mother's arm and the infants' chest recorded cardiac and respiratory responses. Cameras recorded both the mothers' and infants' behavior. Dual functional near‐infrared spectroscopy was also obtained but not analyzed for the present study. [by Gwyneth Wagner].

To measure cardiac synchrony and infants' positive affect, mothers were instructed to play with their infant as they would do at home (Figure 1). We offered them a few toys (e.g., a stacking ring toy, a ball, a doll) and, if they preferred, they could use the toys they brought from home. The interaction continued for 5 min but breaks were given in case the infant got fussy.

Cardiac signals were recorded simultaneously from mother and infant using a portable surface electrocardiogram (ECG) set up (Smarting 24, mBrainTrain) to allow for an unrestricted mobility of the dyad during recordings. We used disposable Swaromed‐ECG‐electrodes (6000063N; https://uk.nisshamedical.com/Deutsch/Electrodes/Swaro.aspx), which were placed below the clavicle and near the right (1) and the left (2) shoulder, on the right (3) and the left (4) lower abdomen, and on the chest (5) as reference. Cardiac signals, sampled at 500 Hz, were monitored and recorded using Smarting Streamer (version 3.3).

#### 2.2.2 Word Segmentation

Infants' ability to segment words was examined with an eye‐tracking based central fixation paradigm (Cooper and Aslin 1990). The stimuli were taken from Bartels et al. (2009). In the original study, half of the infants (group A) were familiarized with passages containing *Balken* (“*balk*”) and *Pinsel* (“*paintbrush*”) whereas half of the infants (group B) were familiarized with the words *Felsen* (“*cliff*”) and *Kurbel* (“*crank*”). Here, we familiarized group A with the passages containing the words *Pinsel* and *Felsen*, and group B with *Balken* and *Kurbel*. In this manner we balanced the stimuli by presenting each group with one target word starting with an early‐pronounced consonant (i.e., *Pinsel* or *Balken*) and the other with a later‐pronounced consonant (i.e., *Felsen* or *Kurbel*). In fact, infants start producing /p/ and /b/ earlier than /k/ and /f/, on average (Sander 1972), and, in language development, the link between perception and production abilities is well‐established (Newman et al. 2006; Tsao et al. 2004; Werker and Yeung 2005). Diverging from Bartels et al., who familiarized infants with auditory word lists and then tested with auditory text passages (i.e., word‐to‐passage order), we familiarized infants with auditory text passages in German containing two target words, and we tested them with auditory novel and familiar words presented in isolation (i.e., passage‐to‐word order). This methodological change was motivated by the fact that familiarization with text passages, compared to word lists, is closer to infants' natural language input. Moreover, this order might bring about larger effects (e.g., Nazzi et al. 2014).

The task began with a familiarization phase during which infants listened to two distinct text passages, repeated twice (i.e., 4 familiarization trials in total). Each text passage contained six sentences, each of which included a target word (i.e., *Pinsel*, *Felsen* or *Balken*, *Kurbel*) at different positions (i.e., at the beginning, in the middle, or at the end of the sentence). Directly after the familiarization phase, all infants were exposed to the same 12 test trials in alternating order. Each test trial played a repeated word, which could be either familiar (i.e., target words presented during familiarization) or novel (i.e., words that were familiarized by the other group of infants). Four distinct test trials appeared three times (i.e., three blocks) in a pseudo randomized sequence. *Pinsel* and *Felsen* were familiar words for group A and novel words for group B, and *Balken* and *Kurbel* were familiar words for group B and novel words for group A. Each word was repeated with diverse prosody for a maximum of 32 s and the number of repetitions was fixed accordingly (i.e., *Balken* repeated 31 times, *Felsen* 27, *Kurbel* 28, *Pinsel* 28).

The familiarization phase started with an attention getter (i.e., a colorful rotating wheel) displayed until the infant fixated on the screen. As soon as the infant focused on the screen, the auditory text passage was played while a colorful checkerboard with blinking squares was shown. A trial lasted until completion, regardless of whether the eye tracker detected infants' eyes on or outside the screen. This scheme was reproduced for each familiarization trial. The test phase started with an attention getter (i.e., colorful rotating wheel) accompanied by a sound (i.e., baby laughter, bike bell, bird song). Once infants looked on the screen, the first test trial, including either a familiarized or a novel repeated word, started while a colorful checkerboard with blinking squares was displayed. Each test trial lasted until completion or until infants looked away for 2 consecutive seconds (i.e., infant‐gaze controlled). As soon as the infants looked back on the screen, the following trial started. If infants looked on the screen longer while listening to novel, as opposed to familiar words, or vice versa, this was taken to indicate that infants segmented words from the auditory text passages.

Looking times were measured with an arm‐mounted EyeLink 1000 eye tracker sampling at 500 Hz with a 16 mm lens. Infants were placed approximately at a 55 cm distance from the eye tracker. Stimuli were displayed on a 21.5 inch BenQ GL2250—LED monitor. Behind the monitor, two Logitech Z200 speakers played the auditory stimuli at an intensity of around 65 dBA. MATLAB (R2018b) was used for programming the experiment. Mothers were asked to place the child in a Maxi‐Cosi in front of the screen. They sat on a chair behind the infant. The following instructions were given to the mothers: not to interact with the child during the experiment, not to point at the screen, and to keep their gaze straight toward the monitor. After a three‐points calibration procedure, the familiarization phase started, followed by the test phase.

#### 2.2.3 Parental Reflective Functioning

We used the Parental Reflective Functioning Questionnaire (PRFQ), a multidimensional self‐report measure (Luyten et al. 2017) with 18 items in total. Each item contained a statement which was rated on a seven‐point Likert scale ranging from 1 (completely disagree) to 7 (completely agree). PRFQ included 3 subscales of six items each. *Pre‐mentalization (PM)* addressed the degree to which parents struggle to understand their children's perspectives and attribute negative intentions to the children's behavior (e.g., “Often, my child's behavior is too confusing to bother figuring out.”). *Certainty about mental states (CMS)* measured how confident parents are in their ability to comprehend their children's thoughts and emotions (e.g., “I can always predict what my child will do.”). *Interest in and curiosity about mental states (IC)* evaluated parents' interest in understanding the mental processes behind their children's actions (e.g., “I wonder a lot about what my child is thinking and feeling.”). The score for each subscale was the mean across the respective six items. Here, we used the global score, summed across all subscales.

### 2.3 Data Processing and Coding

#### 2.3.1 Word Segmentation Task

We extracted the looking time data from EyeLink Data Viewer. The looking time for each test trial was the result of the summed times of the fixations (200 ms) of the right eye on the screen while listening to a given test trial. We discarded test trials with a looking time shorter than 1 s, and we excluded infants who did not provide a minimum of 6 test trials (i.e., three in each condition; cf. Junge et al. 2020).

#### 2.3.2 Cardiac Synchrony

We used NeuroKit (Makowski et al. 2021) to clean the ECG signal and detect r‐peaks. Then, we visually inspected and corrected the data in case of incorrect r‐peak detection on a *react* application (https://react.dev/) using *d3.js* (https://d3js.org/) for visualization through the *visx* library (https://airbnb.io/visx/) (online at https://www.ibxx.at/ibi_v2/). The timing of the detected R‐wave, corresponding to the depolarization of the ventricular myocardium, was used to calculate the Inter‐Beat‐Intervals (i.e., IBIs).

We used *spline interpolation* to infer IBIs in regions that had been excluded in the manual‐correction step. In case the missing IBIs exceeded the overall signal by more than 20%, the participant was excluded from the analysis. The IBIs were then processed according to Abney et al. (2021), with algorithms adjusted for baseline heart rate differences between infants and adults by focusing on RSA magnitude change rates and utilizing distinct frequency bands for RSA detection in infants. Specifically: (1) IBIs were resampled to 5 Hz to account for the different lengths of IBIs in adults and infants (Porges 1986). (2) Band‐pass filters, corresponding to respiratory frequencies, were specific to the age groups, not to individuals. In particular, we used a bandpass filter capturing frequencies from 0 to 1.2 Hz for the mothers and from 0.2 to 1.4 Hz for the infants (see Abney et al. 2021). (3) The length of both mother RSA and infant RSA were set to the length of the mother's resampled IBI through *linear interpolation*. (4) A calculation of sliding windows (length: 15 s, incremented every 200 milliseconds) yielded the logarithm of the variance of RSA samples within each window position. The output was then used as the final continuous RSA data. (5) The continuous RSA was detrended through the MATLAB function “detrend()”.

The cardiac synchrony between mother and infant was then calculated via cross‐recurrence quantification analysis (CRQA). For this, we selected the embedding parameters depending on the point at which the following functions level‐off (Wallot 2018): time delay (T = 30) was selected through the computation of Average Mutual Information (AMI) function; embedding dimensions (*D* = 2) was determined through the computation of False Nearest Neighbor (FNN) function; finally, a threshold parameter (*r* = 0.3) was set so that the resulting average cross RR over that whole sample was < 5%.

CRQA could be considered a complex non‐linear equivalent of cross‐correlation. It is suitable for quantifying non‐stationary coordinative patterns across different modalities and social interactions (Fusaroli et al. 2014). In CRQA, two dynamical systems are represented through cross‐Recurrence Plots (cross RPs (Marwan and Kurths 2002)). CRQA quantifies cross RPs, in terms of how often two time‐series display similar patterns of change or movement, and what the features of the structure of the entrainment between their trajectories are: The selected threshold parameter (*r*) allows for the counting of similar coordinates in the phase‐space as recurrent, while they do not need to be identical (Coco and Dale 2014). CRQA has proven suitable in quantifying multivariate synchrony (e.g., Vanoncini et al. 2022, 2024), particularly cardiac synchrony (Nguyen, Abney, et al. 2021).

Here, we first examined the temporal interdependence of mother and infant RSA through cross RR, namely the degree to which the two RSA trajectories tended to visit similar patterns of change. Second, we measured the predictability of the recurrence structure through ENTR, which captured the predictability of the recurrence between the two RSA trajectories: if the recurrence intervals tended to have the same length, there was high predictability (i.e., low ENTR); if the recurrence intervals had different lengths, there was low predictability (i.e., high ENTR). CRQA was calculated in R with the “crqa” package (Coco et al. 2021).

#### 2.3.3 Infants' Positive Affect

We coded the videos offline for the infants' positive affect using Mangold Interact (version 16) and calculated the proportion of the time spent in positive affect divided by the amount of codable interaction. Following Vanoncini et al. (2022), positive facial expressions were defined as smiles with lips turned upward, and each facial expression needed to last at least 1 s to be coded. Inter‐rater reliability was achieved for 20% (*n* = 6) of the coded dyads (kappa = 0.85).

### 2.4 Statistical Analysis

In RStudio (RStudio Team 2021), we used stepwise linear mixed‐effects regression modeling (using the packages *lme4* and *lmerTest*; Bates et al. 2015; Kuznetsova et al. 2017). The fixed effect structure was determined on the basis of model comparisons relying on the Akaike Criterion (AIC). The dependent variable was looking time in seconds in which the infant looked at the screen during each test trial during the word segmentation task. looking time was not normally distributed, thus we log‐transformed it (Csibra et al. 2016). The independent variable related to the word segmentation task was trial type (sum contrast coded with familiar as +1 vs. novel trials as −1, centering the effects at the grand mean). Other fixed effects were the two continuous predictors, mean centered, reflecting aspects of RSA synchrony, namely cross RR (i.e., the degree to which the two RSA trajectories tended to visit similar states, lower values indicating poor similarity) and ENTR (i.e., the predictability of the recurrence interval's length between the two RSA trajectories, lower values indicating higher predictability). Participants were included as a random effect.

First, we probed whether cross RR (model **1a**) or ENTR (model **1b**) as independent variables concerning cardiac synchrony captured more variance in the looking time data by trial type (familiar vs. novel trials) as compared to a baseline model (model **0**) (Table 1).

**TABLE 1:** Models included in Step 1 of the stepwise linear mixed‐effects regression.

Step 1	
0	${LogLT}_{ij}=\gamma_{00}+\gamma_{10}{TT}_{ij}+u_{0j}+e_{ij}$ *i* = index for trials *j* = index for participants LogLT_*ij*_ = log‐transformed looking time $\gamma_{00}$ = fixed intercept $\gamma_{10}$ = fixed slope TT_*ij*_ = trial type, familiar versus novel $u_{0j}$ = random intercept for participants *j* $e_{ij}$ = residual
1a	${LogLT}_{ij}=\gamma_{00}+\gamma_{10}{TT}_{ij}+\gamma_{20}{RR}_{ij}+\gamma_{30}{TT}_{ij}{\cdot RR}_{ij}+u_{0j}+e_{ij}$ RR_*ij*_ = continuous measure of RSA synchrony indexed by cross RR
1b	${LogLT}_{ij}=\gamma_{00}+\gamma_{10}{TT}_{ij}+\gamma_{20}{ENTR}_{ij}+\gamma_{30}{TT}_{ij}{\cdot ENTR}_{ij}+u_{0j}+e_{ij}$ ENTR_*ij*_ = continuous measure of RSA synchrony indexed by ENTR

Second (Table 2), we included only cross RR or only ENTR, depending on which of the two contributed to explaining more variance in Step 1. For step 2 we explored two different models: model **2a** included the interaction among the three independent variables (trial type, RR/ENTR, PRFQ score); whereas model **2b** included the interactions between trial type and RR/ENTR and trial type and PRFQ, respectively.

**TABLE 2:** Models included in Step 2 of the stepwise linear mixed‐effects regression.

Step 2	
2a	${LogLT}_{ij}=\gamma_{00}+\gamma_{10}{TT}_{ij}+\gamma_{20}{RSA\_Sync}_{ij}+\gamma_{30}{PRFQ}_{0j}+\gamma_{40}{TT}_{ij}\cdot {{RSA\_Sync}_{ij}\cdot PRFQ}_{0j}+u_{0j}+e_{ij}$ RSA_Sync_*ij*_ = continuous measure of RSA synchrony (cross RR or ENTR) PRFQ_0j_ = PRFQ score per participant
2b	${LogLT}_{ij}=\gamma_{00}+\gamma_{10}{TT}_{ij}+\gamma_{20}{RSA\_Sync}_{ij}+\gamma_{30}{TT}_{ij}{\cdot RSA\_Sync}_{ij}+\gamma_{40}{TT}_{ij}+\gamma_{50}{PRFQ}_{0j}+\gamma_{60}{TT}_{ij}{\cdot PRFQ}_{0j}+u_{0j}+e_{ij}$

Third (Table 3), the final full models included infants' positive affect. As for the previous step, model **3a** entailed the full interaction, while model **3b** differentiate each two‐way interaction. Depending on whether the PRFQ score improved the model fit or not, we removed it or left it with the last full model.

**TABLE 3:** Models included in Step 3 of the stepwise linear mixed‐effects regression.

Step 3	
3a	${LogLT}_{ij}=\gamma_{00}+\gamma_{10}{TT}_{ij}+\gamma_{20}{RSA\_Sync}_{ij}+\gamma_{30}{PRFQ}_{0j}+\gamma_{40}{PA}_{0j}+\gamma_{50}{TT}_{ij\;}{\cdot RSA\_Sync}_{ij}{\cdot PRFQ}_{0j}{\cdot PA}_{0j}+u_{0j}+e_{ij}$ PA_0j_ = proportion of positive affect
3b	${LogLT}_{ij}=\gamma_{00}+\gamma_{10}{TT}_{ij}+\gamma_{20}{Rsa\_Sync}_{ij}+\gamma_{30}{TT}_{ij}{\cdot Rsa\_Sync}_{ij}+\gamma_{40}{TT}_{ij}+\gamma_{50}{PRFQ}_{0j}+\gamma_{60}{TT}_{ij}{\cdot PRFQ}_{0j}+\gamma_{70}{TT}_{ij}+\gamma_{80}{PA}_{0j}+\gamma_{90}{TT}_{ij}{\cdot PA}_{0j}+u_{0j}+e_{ij}$

## 3 Results

Overall, cardiac synchrony fluctuated between 2.23 and 3.96 (*M* = 3.04; *SD* = 0.41) for cross RR and between 2.13 and 3.03 (*M* = 2.60; *SD* = 0.19) for ENTR. In the models, we centered both continuous predictors (i.e., cross RR and ENTR). Following model comparisons based on AIC, the most representative model for Step 1 was **1a**, which revealed an AIC value of 666.3, with log‐likelihoods represented in a chi‐square statistic, χ2(2) = 7.864, *p* = 0.02 (for the output of model **1a and 1b**, see Tables 4 and 5, respectively).

**TABLE 4:** Effects of RSA synchrony's cross RR on Looking Times (model **1a**).

	log (looking time)		
*Predictors*	*Estimates*	*CI*	*p*
(Intercept)	1.79	1.64–1.93	**< 0.001**
Trial type	−0.03	−0.10–0.05	0.501
RSA_synchrony_RR	0.38	0.02–0.75	**< 0.05**
Trial type ⋅ RSA_synchrony_RR	−0.20	−0.40 to −0.01	**< 0.05**
**Random effects**			
σ^2^	0.46		
τ_00 Participant_	0.11		
ICC	0.19		
*N*_Participant_	28		

*Note:* Number of observations: 299. Bold values indicate statistical significance.

**TABLE 5:** Effects of RSA synchrony's ENTR on looking times (model **1b**).

	log (looking time)		
*Predictors*	*Estimates*	*CI*	*p*
(Intercept)	1.78	1.63–1.94	**< 0.001**
Trial type	−0.03	−0.10–0.05	0.524
RSA_synchrony_ENTR	0.12	−0.71–0.95	0.772
Trial type ⋅ RSA_synchrony_ENTR	0.04	−0.39–0.47	0.852
**Random effects**			
σ^2^	0.47		
τ_00 Participant_	0.13		
ICC	0.22		
*N*_Participant_	28		

*Note:* Number of observations: 299. Bold values indicate statistical significance.

**FIGURE 2:**
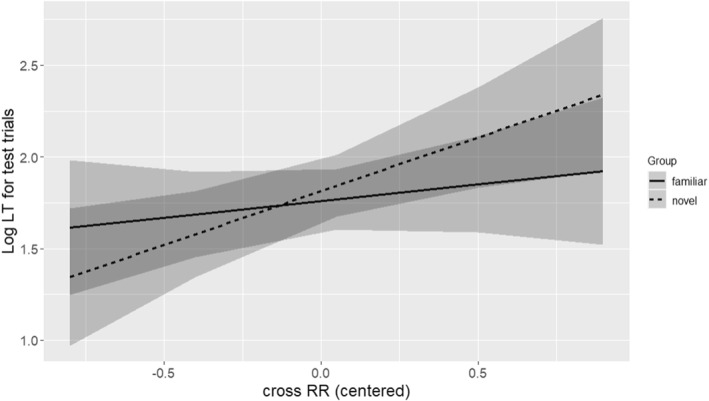
Looking times in the word segmentation test trials by trial type (familiar, novel) as a function of cross RR of cardiac synchrony.

Model **1a** showed that cross RR significantly predicted looking time during the word segmentation test trials with an estimate of 0.38 (*p* < 0.05): the higher the cross RR (i.e., higher amount of mother‐infant cardiac synchrony) during the 5‐min face‐to‐face interaction, the longer infants looked at the screen regardless of the Trial Type (large effect size: Cohen's *d* = 0.79). Moreover, there was a significant interaction between Trial Type and cross RR when predicting looking time with an estimate of −0.20 (*p* < 0.05): the higher the cross RR during the 5‐min face‐to‐face interaction, the longer infants looked at the screen while hearing the novel test words as compared to the familiar test words (small effect size: Cohen's *d* = 0.25; see Figure 2).

Second, we compared model **1a** with models **2a** and **2b**, including the centered PRFQ score as a predictor for the relation of cross RR and Trial Type on looking time. One of the 28 participants did not complete the PRFQ. Overall, the global score of PRFQ fluctuated between 9 and 16 (*M* = 11.55; *SD* = 1.72); with the following scores on the subscales: *pre‐mentalization* (PM) between 1 and 2 (*M* = 1.59; *SD* = 0.50), *certainty about mental states* (CMS) between 2 and 7 (*M* = 4.15; *SD* = 1.26), *parental interest in and curiosity about mental state* (IC) between 5 and 7 (*M* = 5.81; *SD* = 0.79). Neither of the models (**2a** or **2b**) including the PRFQ significantly improved the model fit. However, we noted that the global PRF score was not ideal because an optimal PRF would be characterized by low scores on the *PM* dimension, medium scores on the *CMS* dimension, and medium to high scores on the *IC* dimension. Therefore, we also explored the correlation of each subscale with the selected score for cardiac synchrony (i.e., cross RR). Neither the PM, *r*(25) = 0.18, *p* = 0.36, nor the CMS, *r*(25) = 0.06, *p* = 0.77, subscale correlated with cardiac synchrony. However, the IC subscore showed a significant moderate positive correlation with cardiac synchrony, *r*(25) = 0.42, *p* < 0.05. Thus, we compared model **1a** with models **2a** and **2b**, this time including the centered IC subscore as a predictor for the relation of cross RR and Trial Type on looking time, but neither of the models (**2a** or **2b** with centered IC subscore) significantly improved the model fit when compared to model **1a**.

Finally, for step 3 (model **3a** and **3b**), we added the infants' proportion of positive affect to the model **1a**. For one of the 28 participants affect could not be coded because of the bad quality of the video. Overall, the proportion of positive affect fluctuated between 0 and 0.68 (*M* = 0.18; *SD* = 0.18). Due to the non‐significant contribution of the PRFQ score, we ran models **3a** and **3b** without it (other than indicated in Table 3). Following model comparisons, the large *p*‐value of the full models **3a** (*χ2* (2) = 1.78, *p* > 0.05) and **3b** (*χ2* (2) = 0.07, *p* > 0.05), in addition to the higher AIC (baseline model: 652.30; model **3a**: 658.46; model **3b**: 656.65) indicated that including infants' proportion of positive affect during the face‐to‐face interaction did not improve the model fit above and beyond cross RR and parental reflective functioning.

## 4 Discussion

The current study examined the association between mother‐infant biological coregulation, as indexed by cardiac synchrony, and infant's word segmentation. In addition, it explored whether parental reflective functioning and infants' positive affect relate to this potential link. Our findings support the assumed association between cardiac synchrony during face‐to‐face interaction and infant's word segmentation. Particularly, through regression modeling, we found that the higher the cross recurrence rate (RR) of mother's and infant's RSA, the longer infants look toward the screen while listening to the novel as compared to the familiar test words, which we interpret as advanced word segmentation (DePaolis et al. 2016). However, we found no evidence for (or against) the predictability (i.e., ENTR) of the length of the concurrent RSA intervals to be related to infant's word segmentation. Moreover, we found no evidence for or against the possibility that this association was enhanced by higher parental reflective functioning skills, or by a higher degree of infants' positive affect during face‐to‐face interaction. In the following sections, our results are discussed in relation to previous research.

The 9‐month‐olds showed a more advanced word segmentation performance when they experienced higher cardiac synchrony with their mothers during face‐to‐face interaction. Cardiac synchrony was measured through RSA, which reflects the parasympathetic activity (Camm et al. 1996). This finding is consistent with research showing that infants require their caregiver to stabilize their emotions, behavior, and physiology on a daily basis (Buhler‐Wassmann and Hibel 2021). Moreover, free play represented one of the common parent‐infant interactive activities in which we can observe coregulation, and mothers synchronize their RSA based on their infant's regulatory capacities (Lunkenheimer et al. 2021).

We reasoned that the link between interpersonal synchrony and language development concerned two main directions. First, language as a tool could lead to social coordination (Tylén et al. 2010). Therefore, infants who are more advanced in their word segmentation performance might also be more advanced in their language abilities in general and in sharing meaning through one of the first forms of communication (i.e., coregulation). This, in return, might lead to higher synchrony when playing and interacting with their primary caregiver. Second, synchrony, which has been argued to reflect the quality of the interaction by sustaining the emotional balance, could provide a safe and conducive environment (Beebe et al. 2010; Feldman 2003), and this type of positive environment might support language development (Harrist and Waugh 2002), in this case word segmentation.

Our results show that the association between language development and coregulation can be seen not just at the emotional, behavioral and neural (Vanoncini et al. 2022, 2024), but also at the physiological level. About this association, a previous study has found that parent‐child physiological synchrony is associated with coordinated behavioral and emotional responsivity (Feldman 2012). Our findings are in line with the studies showing that fluent and connected communication, regardless of whether it is verbal or nonverbal, is linked with infant's attention and language development (Masek, Paterson, et al. 2021). On the one hand, fluent and connected communication allows infants to understand the communicative intent. On the other hand, infant's attention reduces referential ambiguity in input and link word to referent (Tamis‐LeMonda et al. 2014). In fact, language development might be the result of the intertwined relation between behavioral caregiver‐infant contingency (e.g., vocal contingency) and infant attention: contingency facilitates the development of infant attention and, in turn, infant attention enhances participation in contingent interactions (Masek, McMillan, et al. 2021). Similar results come from a recent study indicating that synchrony, both behavioral and physiological, may increase infant's receptivity to language stimulation (McFarland et al. 2020), providing a scaffolding for language development. Future research should aim to examine the mediating or moderating role of attention when linking synchrony and language development.

We investigated whether two aspects of interpersonal synchrony at the physiological level—dyadic coupling (i.e., recurrence) and dyadic predictability (i.e., entropy) of RSA—as a reflection of coregulation are linked to language development. Dyadic coupling captured the amount of coregulation during the face‐to‐face interaction; whereas dyadic predictability indexed how predictable the interval lengths of dyadic coupling was. One point was in contrast with previous studies: both Vanoncini et al. (2022),  (2024) found that the predictability of the interval lengths of mother‐infant synchrony related to infant's word segmentation, while here we found that recurrence rather than predictability of interval lengths showed that effect. This might be due to the difference in the modalities of synchrony examined in the three studies: while Vanoncini et al. (2022),  (2024) examined behavioral and neural aspects of coregulation, namely facial expressions and gaze‐infant's social brain, respectively; here, we study cardiac synchrony as a reflection of biological synchrony. Possibly, 9‐month‐olds might be sensitive to behavioral predictabilities in interactions, but not to covert aspects such as the heart beats (in this case RSA). Moreover, the difference might be due to the nature of the cardiac versus neural signals: the former originate from the heart and regulate its activity in a highly regular way; the latter arise from the nervous system and regulate several bodily functions through higher fluctuations and dynamics.

The present study did not find evidence that overall parental reflective functioning was relevant to infant's word segmentation, or to the link between infant's word segmentation and cardiac synchrony. One reason for this null finding might have concerned the multidimensionality of the PRFQ and their different interpretations, as optimal PRF would be expressed by low *PM*, medium *CMS*, and medium to high *IC* scores in the subtest; however, our explorations of the individual subtest scores as moderators did not result in significant effects either. Another reason may relate to the low diversity in our reduced sample: the overall scale might not be sensitive enough if study samples do not include clinical groups or individuals from a high‐risk context. A further potential reason is that parental estimates might not be reliable: mothers might not be fully aware of their biological responsiveness to their infants, as parental reflective functioning requires an awareness of their own and their child's internal mental states. Ideally, future studies could obtain additional more objective observational measures (e.g., Lee et al. 2023). Finally, our findings could support the notion of parental embodied mentalizing (Shai and Belsky 2011): As verbal communication can often be insufficient for understanding and responding to a child's emotional and developmental needs, parents' unconscious embodied responses, like cardiac attunement, might play a crucial supportive role in parent‐child interactions.

The current investigation did not find evidence for an effect of infants' positive affect on the link between cardiac synchrony and infants' word segmentation. This is in line with the mixed findings reported so far: Some studies have shown that cardiac synchrony increases during moments of positive affect and simultaneous positive vocalizations (Feldman et al. 2011), whereas others found cardiac synchrony in the presence of infant's negative affect (Nguyen, Abney, et al. 2021; Wass et al. 2019). Differences in participants' age, type of measure and method, and selected CRQA metrics might explain our null‐finding, together with the fact that in the current study, there were few occurrences of negative affect (the task was a face‐to‐face interaction with free play), as well as that our cardiac synchrony measure subsumed both positive (i.e., both individuals increase or decrease their RSA) and negative directions (i.e., one individual decreases while the other increases their RSA). Future studies may aim to capture cardiac synchrony in relation to different facial expressions as a measure of their emotional state.

This study has several limitations. It was part of a large‐scale project on mother‐infant synchrony, collecting longitudinal data from 90 dyads at 9 and 18 months. Each session included six paradigms lasting approximately 3 hours (including breaks), employing a multilevel methodology in a naturalistic setting. The high dropout rate likely stemmed from challenging testing conditions, especially in the context of hygiene restrictions during the COVID‐19 pandemic. Using dual electrocardiography for simultaneous recording of mothers' and infants' activities added complexity, as some infants were uncomfortable with the electrodes despite being non‐invasive. High‐quality data from both the mother and infant is essential for examining synchrony, and we lost 18 dyads because of poor ECG data from either the infant or the mother. Furthermore, naturalistic interactions increased the probability of motion artifacts, despite our efforts to mitigate them. Finally, the counterbalanced task order across dyads may have contributed to higher data loss for dyads who received the current tasks as their final ones. The high dropout rate may reflect a sample bias, necessitating cautious interpretation of our findings and raising concerns about temperament biases. To address this, caregiver data from the Infant Behavior Questionnaire‐Revised (IBQ‐R VSF; Putnam et al. 2014) were analyzed. Of the 81 caregivers, 86% completed the IBQ‐R, revealing no significant differences in reported infant temperament between participants (*n* = 27) and dropouts (*n* = 43). However, the “positive affectivity/surgency” subscale was significantly higher in excluded (vs. included) infants, suggesting it may have contributed to the increased dropout rate, potentially impacting the ECG data quality and findings' generalizability. Future studies using this challenging technology and design should expand the sample size to increase the statistical power and generalizability of the results, and implement strategies (e.g., shorter test sessions) to reduce dropouts and improve participant retention.

Taken together, this paper examined how mother‐infant cardiac synchrony relates to language development. Our findings of an association between biological coregulation and advanced word segmentation contribute to the understanding of biological and social factors that influence early language development. Remarkably, synchronized social interactions, starting from their physiological level, might have a positive impact on communication effectiveness and the creation of an interconnected environment for language growth. Further research is needed to explore the potential mechanisms underlying the observed association and to investigate additional factors that may influence the relationship between cardiac synchrony and infant word segmentation.

## Author Contributions

**Monica Vanoncini:** conceptualization, data curation, formal analysis, investigation, methodology, project administration, visualization, writing – original draft, writing – review and editing. **Ezgi Kayhan:** conceptualization, data curation, funding acquisition, methodology, project administration, supervision, writing – review and editing. **Birgit Elsner:** funding acquisition, supervision, writing – review and editing. **Moritz Wunderwald:** software, writing – review and editing. **Sebastian Wallot:** formal analysis, writing – review and editing. **Stefanie Hoehl:** conceptualization, methodology, resources, software, writing – review and editing. **Natalie Boll‐Avetisyan:** conceptualization, funding acquisition, methodology, supervision, writing – review and editing.

## Data Availability

The data that support the findings of this study are openly available in OSF at http://doi.org/10.17605/OSF.IO/S9ZEB. The preregistration is available at the following URL: https://aspredicted.org/KHR_36Y.
